# Safety and efficacy of direct-acting antivirals for chronic hepatitis C in patients with chronic kidney disease

**DOI:** 10.1186/s12882-020-1687-1

**Published:** 2020-01-16

**Authors:** Elena Laura Iliescu, Adriana Mercan-Stanciu, Letitia Toma

**Affiliations:** Department of Internal Medicine II, Fundeni Clinical Institute, 022328 Bucharest, Romania

**Keywords:** Hepatitis C virus, Direct-acting antiviral therapy, Chronic kidney disease, Kidney transplantation

## Abstract

**Background:**

This is a real-world evidence study that aims to analyze the efficacy, tolerability and safety profile of paritaprevir/ombitasvir/ritonavir and dasabuvir, in patients with renal impairment.

**Methods:**

We conducted an observational prospective study, on 232 patients with chronic kidney disease, undergoing treatment with paritaprevir/ombitasvir/ritonavir and dasabuvir, for chronic hepatitis C infection - genotype 1b. Renal and liver function were assessed at the beginning of therapy, monthly during treatment and three months after therapy completion.

**Results:**

All patients achieved sustained virologic response. Common side effects were nausea, fatigue and headache. Close monitoring of tacrolimus blood levels and dose reduction was required in kidney transplant recipients.

**Conclusions:**

HCV therapy in the setting of renal dysfunction has always been a challenging topic. Direct-acting antivirals have shown promising effects, demonstrating good tolerance and efficacy in patients with HCV infection and renal impairment. Sustained virologic response within our study population was 100%.

## Background

Hepatitis C virus (HCV) affects over 70 million people worldwide, corresponding to 1.0% of the global population [1]. Public interest in HCV is growing, especially since the virus can also induce extrahepatic manifestations (in 40–70% of cases) including autoimmunity-related symptoms, metabolic, renal, cardiovascular, central nervous system or lymphoproliferative disorders [2, 3].

Renal complications often appear in the context of cryoglobulinemia [4]. Due to the persistence of the virus in the organism, an overstimulation of B lymphocytes occurs, leading to the production of mixed cryoglobulins (a group of globulins with the property of reversible precipitation at low temperatures) [5]. Recently, toll-like receptors (TLRs) – important components of the innate immune system, have been attributed a role in HCV-associated renal injury, due to their ability to recognize molecular patterns related to microbial structures and, thus, to induce an immune response [5, 6].

Chronic HCV infection exacerbates renal deterioration, resulting in end-stage renal disease (ESRD) and increasing morbidity and mortality in patients undergoing dialysis, as well as in kidney transplant recipients (KT) [3]. Therefore, HCV therapeutic approach has always represented a challenging topic. Interferon-based therapies have demonstrated frequent and severe side-effects [7–9]. The development of direct acting antivirals (DAAs) has led to a new era, showing promising effects, even in patients with chronic kidney disease (CKD) [7–9].

Concerning the combination paritaprevir/ombitasvir/ritonavir and dasabuvir, studies have shown good tolerance and efficacy in HCV-infected patients with stage 4/5 CKD, including subjects with compensated cirrhosis, previously treated patients and patients undergoing dialysis [10]. We evaluated the safety and efficacy of this therapeutic regimen in a group of 232 patients, including subjects with severe renal impairment, hemodialyzed patients and kidney transplant recipients, all infected with HCV genotype 1.

## Methods

We conducted an observational prospective study on 232 patients*,* infected with HCV genotype 1b, who received interferon-free treatment with paritaprevir/ombitasvir/ritonavir and dasabuvir, for 12 weeks. The subjects were admitted to our clinic between May 2017 and December 2018 and presented different forms of renal disease (including renal transplantation and hemodialysis). During this period, only 4 patients referred to our clinic were considered ineligible for the antiviral therapy - due to the presence of different types of malignancies that required specific oncological treatment.

The study was approved by the Institutional Board of Fundeni Clinical Institute. An informed written consent was obtained from all the subjects. All the records were confidential. The main objective of this study was to evaluate the effectiveness and safety of HCV treatment in CKD population.

All patients received DAAs therapy for 12 weeks. Regarding the combination containing 12.5/75/50 mg of ombitasvir (OBV), paritaprevir (PTV) and ritonavir (r), each patient was instructed to ingest two tablets daily, in the morning, with food, for maximal absorption of the active substances. The recommended dose of dasabuvir (DSV) was 250 mg (one tablet), twice a day, in the morning and evening, during meals. Patients with renal replacement therapy were instructed to take the medication as earlier as possible, in the days with dialysis scheduled in the afternoon.

The degree of fibrosis was assessed by both Fibromax and Fibroscan, before initiating the treatment. The results of the tests were concordant: 139 patients with F2 fibrosis stage, 74 patients with F3 and 19 patients with F4 (cirrhosis). Due to potential complications, no patient underwent liver biopsy for evaluation. In patients undergoing hemodialysis, non-invasive fibrosis tests represent a satisfactory alternative in the assessment of hepatic fibrosis [11].

Viral infection was evaluated by quantitative HCV ribonucleic acid (RNA) determination, at the beginning of therapy, at the end of treatment (EOT) and at 12 weeks after EOT, using the Roche COBAS® Ampliprep TNAI/TaqMan® 48 RUO Assay for HCV RNA Quantification for HCV in human serum or EDTA plasma samples. The follow-up time for each patient was 24 weeks. Patients were considered to have achieved sustained virologic response (SVR) only if they had HCV-RNA levels under the lower limit of quantification, at both EOT and week 12 post-treatment.

Subjects’ evaluation included abdominal ultrasonography at the beginning (week 0), at the end of treatment (week 12), as well as 3 months after completing therapy (week 24). Signs and symptoms were assessed monthly through history and clinical examination.

Biological tests included assessment of liver enzymes, bilirubin, blood urea nitrogen, serum creatinine, uric acid, hemoglobin and a 24-h urine protein test, which were periodically determined at weeks 0, 4, 8, 12 and 24 in all patients. Also, in some selected patients (with autoimmune disorders or diabetes), C3 and C4 fractions of the complement, rheumatoid factor, erythrocyte sedimentation rate, C reactive protein, serum cryoglobulins, glucose and hematuria were assessed. A notable exception was represented by the hemodialyzed subjects; in their case, the evolution of serum creatinine was considered inaccurate, therefore, this parameter was not determined. Estimated glomerular filtration rate (eGFR) was calculated with CKD-EPI equations. In kidney transplant recipients, blood levels of tacrolimus were strictly monitored.

## Results

Data was prospectively collected and analyzed from a cohort of 232 patients, infected with HCV genotype 1B and with renal impairment. Of these, 5.1% (12 subjects) were kidney transplant recipients, while 20.7% (48 subjects) were undergoing hemodialysis. Most patients, 47.8% (111 individuals) had stage 2, 3 or 4 CKD (diabetes mellitus or arterial hypertension related). A percentage of 24.1% (56 subjects) presented evidence of mixed cryoglobulinemia, while 2.1% (5 patients) had developed renal disease in the context of systemic lupus erythematous. Assessment of fibrosis degree resulted in 139 F2 patients (59.9%), 74 F3 patients (31. 9%) and 19 F4 individuals (8.2%). The mean age was 58.3 +/− 11.9. Most subjects (54.7%) were females, with no significant age difference between genders. The follow up time was 24 weeks. Further baseline characteristics are presented in Table 1.

**Table 1:** Baseline characteristics of the study group

Mean age	58.29 +/− 11.9 years
Sex ratio	Males: 105 (45.2%) Females: 127 (54.7%)
Mean HCV- RNA	1,298,674 +/− 725,624 IU/ml
F2 fibrosis	139 patients (59.9%) - 48 patients with cryoglobulinemia - 4 patients with SLE - 54 patients with CKD stage 2/3 (diabetes mellitus related, arterial hypertension related) - 28 patients undergoing hemodialysis - 5 patients after kidney transplantation
F3 fibrosis	74 patients (31.9%) - 5 patients with cryoglobulinemia - 1 patient with SLE - 49 patients with CKD stage 2/3 (diabetes mellitus related, arterial hypertension related) - 12 patients undergoing hemodialysis - 7 patients after kidney transplantation
F4 fibrosis	19 patients - 3 patients with cryoglobulinemia - 8 patients with CKD stage 2/3 (diabetes mellitus related, arterial hypertension related) - 8 patients undergoing hemodialysis
Median ALT level	35 U/L (range 15 U/L – 217 U/L)
Median bilirubin level	1.2 mg/dL (range 0.5 mg/dL – 2.8 mg/dL)
Mean hemoglobin level	12.9 +/− 4.2 g/dL

### Results in HCV-infected population presenting cryoglobulinemia

Cryoglobulinemia was defined based on the presence of cryoglobulins in the blood of the patients. Amongst these patients, women were predominant (64.2%). Most of the cryoglobulinemic population had fibrosis F2 (85.7% - 48 individuals), while 8.9% (5 patients) were assessed as being F3; a low percentage of 5.3% (3 subjects) had cirrhosis (Table 2). The median declared duration of viral infection was 7 years. The modality of contracting the infection remained unknown.

**Table 2:** Baseline characteristics in each study group

	Patients with cryoglobulinemia (*N* = 56)	Patients with SLE (N = 5)	Patients with CKD (hypertensive or diabetic) (*N* = 111)	Patients undergoing hemodialysis (*N* = 48)	Patients after kidney transplant (*N* = 12)
Gender	M: 20 pts. (35.8%) F: 36 pts. (64.2%)	M: 0 pts. F: 5 pts. (100%)	M: 51 pts. (46%) F: 60 pts. (54%)	M: 27 pts. (56.2%) F: 21 pts. (43.8%)	M: 7 pts. (58.3%) F: 5 (41.6%)
Fibrosis	F2: 48 pts. (85.7%) F3: 5 pts. (8.9%) F4: 3 pts. (5.3%)	F2: 4 pts. (80%) F3: 1 pts. (20%) F4: 0 pts	F2: 54 pts. (48.6%) F3: 49 pts. (44.1%) F4: 8 pts. (7.2%)	F2: 28 pts. (58.3%) F3: 12 pts. (25%) F4: 8 pts. (16.6%)	F2: 5pts (41.6%) F3: 7 pts. (58.3%) F4: 0 pts
Stage of CKD	Stage 2: 13 pts. (23.2%) Stage 3: 39 pts. (69.6%) Stage 4: 4 pts. (7.1%)	Class 2 nephritis: 3 pts. (60%) Class 3 nephritis: 1 pts. (20%) Class 4 nephritis: 1 pts. (20%)	Stage 2: 62 pts. (55.8%) Stage 3: 31 pts. (27.9%) Stage 4: 18 pts. (16.2%)		Stage 2: 0 pts. Stage 3: 12 pts. (100%) Stage 4: 0 pts
Hemglobin	Normal: 7 pts. (12.5%) Mild anemia: 49 pts. (87.5%)	Normal: 2 pts. (40%) Mild anemia: 3 pts. (60%)	Normal: 103 pts. (92.8%) Mild anemia: 8 pts. (7.2%)	Normal: 0 pts. Mild anemia: 29 pts. (60.5%) Moderate anemia: 19 pts. (39.5%)	Normal: 8 pts. (66.6%) Mild anemia: 4 pts. (33.3%)

Most patients (50–89.3%) presented palpable purpura of the lower legs. Almost a third of these patients (15 subjects) complained of arthralgia, asthenia, muscle pain, while in 4 cases (7.1% of all cryoglobulinemic patients), the purpuric lesions had already progressed to chronic ulcerations. Neurological involvement, manifesting with painful paresthesia in the lower limbs, was described by 5 patients (8.9%), including those with ulcerative lesions. However, there were 6 patients (10.7%) that presented no signs of cutaneous involvement.

The mean eGFR was 55.48 mL/min/1.73 m^2^ (ranging between 27 and 81 mL/min/1.73 m^2^). Most of the patients had stage 3 CKD (39 patients - 69.6%); 13 individuals (23.2%) had stage 2 CKD and 4 patients had stage 4 CKD (7.1%) (Table 2). Serum creatinine varied from 1.1 to 2.7 mg/dl, at the beginning of treatment. The median cryocrit level was 3.0%.

Before treatment, liver enzymes (ALT, AST) were slightly increased in 12 patients (21.4%), while bilirubin was normal in all patients. Mild anemia was present in 49 patients (87.5%), with hemoglobin values between 11 and 12.6 g/dl. Only 7 patients (12.5%) had normal hemoglobin (Table 2). Rheumatoid factor was present in 44 patients (78.5%), while 49 patients (87.5%) had low levels of complement factions.

Renal involvement of HCV associated cryoglobulinemia was characterized by the following findings: microscopic hematuria was present in 25 patients (44.6%); proteinuria was present in all subjects, with daily protein excretion below 500 mg in 27 patients (48.2%), daily protein excretion between 0.5 and 3.5 g in 20 patients (35.7%) and nephrotic range values of proteinuria in 9 patients (16.1%).

During treatment, no patient experienced significant worsening of kidney or liver function. Transitory increased levels of liver enzymes were observed in 20 patients (35.7%) at week 4 evaluation, including those 12 patients that had already high transaminase levels at the beginning of therapy, but with spontaneous resolution and normal values at week 8 evaluation. Also, the blood tests performed at week 4 of treatment, showed slight elevation of bilirubinemia (up to 2 mg/dl) in 4 individuals (7.1%). These patients received ursodeoxycholic acid, followed by normalization of this parameter by week 8. Hemoglobin values did not differ significantly from the baseline levels during treatment (*p* = 0.16).

Notably, DAAs use decreased the levels of proteinuria in all HCV patients with cryoglobulinemia (*p* = 0.013). Furthermore, proteinuria was dramatically decreased after the first month of therapy in 4 of the 9 patients presenting nephrotic syndrome. These patients received no other drugs concomitantly. Positive results were also noticed in terms of remission of hematuria in all patients, by the end of treatment.

Mild side-effects such as asthenia, nausea or headaches were reported during therapy (Table 3). No patient discontinued or interrupted the antiviral treatment, resulting in undetectable values of HCV-RNA at the end of treatment, as well as SVR. HCV eradication also resulted in the disappearance of serum cryoglobulins in 29 patients (51.7%), while the other 48.2% only presented with a variable decrease in cryoglobulin circulating levels (*p* = 0.026). Complete clinical response and disappearance of purpuric lesions were noted in 47 patients (83.9%). However, 5 patients with cutaneous manifestations and nephrotic range proteinuria at the beginning of therapy (8.9%) only showed partial clinical improvement at SVR; notably, all 5 patients also had neurological involvement with paresthesia and 4 of them also presented ulcerative lesions. Another 4 patients with nephrotic syndrome (7.1%) presented no clinical response, in spite of achieving SVR and improving renal function.

**Table 3:** Adverse reactions to DAAs in the study groups

	Patients with cryoglobulinemia (N = 56)	Patients with SLE (N = 5)	Patients with CKD (hypertensive or diabetic) (N = 111)	Patients undergoing hemodialysis (N = 48)	Patients after kidney transplantation (N = 12)
Asthenia/ Fatigue	10 pts. (17.8%)	2pts (40%)	50 pts. (45%)	7 pts. (14.5%)	2 pts. (16.6%)
Nausea	8 pts. (14.2%)	0 pts	22 pts. (20%)	12 pts. (25%)	3 pts. (25%)
Headache	17pts (30.3%)	2 pts. (20%)	36 pts. (33%)	8 pts. (16,6%)	0 pts
Diarrhea	2 pts. (3.5%)	0 pts	7 pts. (6.3%)	3 pts. (6.2%)	1 pts. (8.3%)
Dizziness	12 pts. (21.4%)	1 pts. (20%)	41 pts. (36.9%)	10 pts. (20.8%)	1 pts. (8.3%)

Normal hemoglobin values were noticed in all patients, at three months after completing therapy (*p* = 0.019).

### Results in HCV-infected population diagnosed with systemic lupus erythematous (SLE)

The study included 5 women with SLE. Four of them (80%) had F2 fibrosis and one female had F3 (Table 2). The median declared duration of viral infection was 4 years; the modality of contracting the infection remained unknown.

There were 3 patients (60%) with class 2 lupus nephritis (pure mesangial alterations), 1 patient (20%) with class 3 lupus nephritis (focal segmental glomerulonephritis, affecting less than 50% of the glomeruli) and 1 patient (20%) with class 4 lupus nephritis (diffuse glomerulonephritis), with severe proteinuria (4.4 g/ 24 h) (Table 2).

The use of immunosuppressive therapies in all patients with lupus was discontinued during DAAs treatment, due to the potential interactions between the two categories of drugs. However, despite the cessation of immunosuppression, significant decreases in proteinuria have been noticed, as early as week 4, especially in patients who had previously answered mildly to immunosuppression (*p* = 0.022). The patient with class 4 lupus nephritis presented significantly lower values of daily protein excretion; after the first month of treatment, proteinuria decreased from 4.4 to 1.6 g/24 h, continuing the descending pattern and reaching 0.7 g/24 h at EOT and 0.5 g/24 h at SVR. There was also a notable decrease in serum inflammatory markers in all 5 patients (*p* = 0.019). All patients reached sustained virologic response.

### Results in HCV-infected population with CKD due to diabetic or hypertensive nephropathy

This group included 111 patients, with a slight predominance of females (54%). There were 54 patients with F2 fibrosis (48.6%), 49 patients with F3 (44.1%) and 8 patients with F4 (7.2%) (Table 2). All patients had comorbidities such as diabetes mellitus and arterial hypertension: 23 patients (20.7%) were both diabetic and hypertensive, 61 patients (54.9%) had only been diagnosed with arterial hypertension, while 27 patients (24.3%) only suffered from diabetes mellitus. The median declared duration of viral infection was 7 years (range 2–23 years). The infecting modality remained unknown.

The mean age was 59.2 +/− 10.7. The mean eGFR was 62 mL/min/1.73 m^2^: more than half of the patients presented stage 2 CKD (55.8%), while 31 individuals (27.9%) had stage 3 CKD and 18 patients (16.2%) were diagnosed with stage 4 CKD (Table 2). Serum creatinine varied between 1.1 and 2.7 mg/dl.

Among the 50 diabetic patients, 7 (14%) had type 1 diabetes mellitus, while the rest of them (86%) were diagnosed with type 2 diabetes. Also, a significant proportion (38%) had poorly-controlled diabetes, with high levels of glycosylated hemoglobin (6.7–8.1%). The mean value of fasting blood glucose at the beginning of therapy was 136 mg/dl.

Most of the 84 hypertensive patients had stage 3 arterial hypertension (82.1%), while the rest of them (17.8%) suffered from stage 2 arterial hypertension.

Liver enzymes presented high values in 25 patients (22.5%), while bilirubin was within normal limits in all subjects. Hemoglobin values were normal in most cases (92.8%). Only a low percentage of patients (7.2%) had mild anemia. Proteinuria was present in almost 19% of the patients, with levels under 500 mg per day.

One month after beginning therapy, liver function improved for those subjects that presented high transaminase levels, achieving normalization of these parameters. Mild adverse effects (nausea, asthenia, headaches) were also observed within this group, with spontaneous resolution after ending the treatment (Table 3). The values of hemoglobin (*p* = 0.215), serum creatinine (*p* = 0.197), eGFR (*p* = 0.268) and proteinuria (*p* = 0.323) did not differ significantly from the baseline levels. Notably, all patients with poorly-controlled diabetes achieved a satisfying glycemic control by the end of treatment (*p* = 0.017), while most insulin-dependent subjects required a decrease in daily insulin doses. All patients had undetectable values of HCV-RNA at the end of treatment, achieving SVR.

### Results in HCV-infected population undergoing hemodialysis

We observed 48 patients undergoing hemodialysis, mostly males (27 subjects – 56.2%). Within this group, there were 28 individuals with F2 (58.3%), 12 patients with F3 (25%) and 8 subjects with F4 (16.6%) (Table 2). No patient had hepatitis B virus (HBV) or human immunodeficiency virus (HIV) coinfection. The mean age was 48.9 years. The mean duration of hemodialysis therapy was 2.8 years. The median declared duration of viral infection was 7 years (range 2–24 years). The modality of contracting the infection remained mostly unknown.

Liver enzymes were increased in 10 patients (20.8%), at the beginning of therapy, while bilirubin was normal in all subjects. Another 5 patients presented asymptomatic high transaminase levels within the first month of treatment, but all subjects showed normal liver enzymes at week 8 evaluation (*p* = 0.026). Mild anemia was encountered at the beginning of treatment in 29 patients (60.5%), while the rest had moderate anemia (39.5%) (Table 2). The hemoglobin values did not differ significantly from the baseline levels, during or after treatment (*p* = 0.281). Most of the adverse effects were mild or moderate, including fatigue, diarrhea, nausea, headache and dizziness. No severe reactions were noted (Table 3).

All patients had undetectable values of HCV-RNA at the end of treatment, achieving SVR.

### Results in HCV-infected population that underwent kidney transplant

Only 12 subjects (5.2%) were kidney transplant recipients. Most of them were males (58.3%). Within this category of patients, there were no cases of cirrhosis, but only F3 (7 patients – 58.3%) and F2 fibrosis (5 patients – 41.6%) (Table 2). All kidney transplant recipients were on tacrolimus-based immunosuppressive therapy, in dosages particularized for each individual, under the appropriate surveillance of a nephrologist specialized in kidney transplant follow-up. Blood measurements performed before initiating antiviral therapy showed tacrolimus levels within the therapeutic range (5–10 ng/ml) in all patients. However, immunosuppressive therapy had to be discontinued, due to possible drug interactions. Tacrolimus doses were lowered to 2 mg/week and closely monitored, as recommended by the guidelines.

No subject had HBV or HIV coinfection. The mean age was 45.8 years. The median declared duration of viral infection was 8 years (range 2–25 years), although, for most of them, the modality of contracting the infection remained unknown. Most of the patients (> 80%) were treatment-naïve.

The mean eGFR was 49 mL/min/1.73 m^2^: all patients had stage 3 CKD (with variations of eGFR between 37 and 59 mL/min/1.73 m^2^). Serum creatinine was between 1.55 and 1.9 mg/dl. (Table 2).

At the beginning of therapy, all patients had normal liver enzymes and normal bilirubin. Only 4 patients (33.3%) suffered from mild anemia (with hemoglobin values between 11.3 and 12.5 g/dl in both men and women), while the rest of them (8 patients – 66.6%) had normal hemoglobin (Table 2). Proteinuria was present in only 2 patients (16.6%), with daily protein excretion below 500 mg.

During therapy, no patient experienced worsening of kidney or liver function. Mild adverse effects were reported, with spontaneous resolution after ending the treatment (Table 3). The values of hemoglobin and proteinuria did not differ significantly from the baseline levels (*p* = 0.219 and respectively *p* = 0.331).

Regarding the efficacy of DAAs, we noticed that all kidney transplant recipients had undetectable values of HCV-RNA at EOT, as well as 12 weeks after EOT. None of the cases required interruption of the therapeutic regimen.

A particular situation is the case of a male patient who underwent two kidney transplants. Due to a history of chronic glomerulonephritis during childhood, leading to CKD stage 5 and hemodialysis, he had the first parent-to-child kidney transplant by the age of 25. Years after the procedure, he was diagnosed with chronic hepatitis C, for which he had received interferon-based therapy, with good outcome and undetectable viremia at the end of treatment (but with no SVR achievement). The patient suffered chronic renal allograft rejection and underwent a second kidney transplantation at the age of 40, followed by viral reactivation. When admitted to our clinic, the Fibromax evaluation revealed F3 fibrosis, while eGFR was 41 ml/min/1.73 m^2^ (CKD Stage 3). He was undergoing daily immunosuppressive therapy with tacrolimus (2 mg/day), mofetil mycophenolate (1 mg/day) and prednisone (5 mg/day). Before initiating DAAs, the subject was instructed to diminish the tacrolimus intake to 2 mg/week; the doses of mofetil mycophenolate and prednisone were not changed. However, the patient accidentally overlapped the two therapies (DAAs and tacrolimus) for 2 days at the beginning of treatment. On week 3, he presented to the hospital with severe hyperglycemia (> 700 mg per dl) and high tacrolinemia (> 30 ng/ml). Intensive insulin protocol was applied and tacrolimus administration was temporarily stopped, until normalization of blood levels. The patient did not present kidney failure or neurologic symptoms. During this period, interferon-free treatment was maintained, resulting in SVR.

## Discussion

Hepatitis C treatment in the setting of renal impairment has always represented a challenging issue. The use of interferon-based products has set various barriers, when referred to HCV-infected patients with CKD, from dose-adjustment questions to severe adverse effects and low efficacy rates [12–14]. However, when achieved, the viral clearance has been correlated with various benefits and improvement in patients presenting CKD [15–17].

It has nowadays been demonstrated that therapeutic regimens based on DAAs are safe and efficient in HCV infected individuals. Nevertheless, tolerance, safety and effectiveness among patients with different degrees of renal impairment deserve further inquiry, as this particular issue remains poorly characterized and understood. The aim of this paper was to assess the efficacy, tolerability and safety profile of paritaprevir/ ombitasvir/ ritonavir and dasabuvir, in HCV patients with CKD.

An important aspect of HCV infection is represented by its association with mixed cryoglobulinemia and, therefore, with membrano-proliferative glomerulonephritis (MPGN): 90% of the patients presenting mixed cryoglobulinemia are HCV-positive, while 50% of the population diagnosed with chronic hepatitic C have circulating cryoglobulins. The histopathological pattern associated with this condition is represented by MPGN (in approximately 80% of the cryoglobulinemic patients) [18].

Within the 232 patients we observed, there were 56 cases of cryoglobulinemia, with a predominance of women, most of them categorized as F2 and F3, with only a very low percent of cirrhotic patients. All patients achieved SVR, as none of them necessitated discontinuation of therapy. For half of the patients, the presence of cryoglobulins was no longer detected in the blood samples, a pattern that is persistent with data reported in other medical studies [19]. Also, more than 80% of the patients presented complete clinical response. Permanent disappearance of symptoms and serum cryoglobulins were also reported in an HCV case treated with ombitasvir/ paritaprevir/ ritonavir, dasabuvir and ribavirin [20]. However, in our case, approximately 16% of the subjects had partial or no improvement of the cutaneous lesions. The factors associated with poor response included severe forms of cryoglobulinemic vasculitis and peripheral neuropathy. A strong dissociation between virologic and clinical responses in patients with HCV infection and cryoglobulinemia has been described by Sollima et al. [21], and possibly explained by a variety of factors such as: severe forms of cryoglobulinemic vasculitis (a situation that, as mentioned, also applies for the partially responsive or non-clinically-responsive patients observed in our study), delayed clearance of circulating cryoglobulins (by comparison to the viral clearance) and/or a partial suppression regarding the proliferation of B cells, resulting in cryoglobulin production that may not be entirely triggered by the HCV. It is suggested, therefore, that a longer follow-up should be performed for these patients, in order to better define their outcome, after achieving SVR [21].

Regarding the interferon-free therapy with paritaprevir/ ombitasvir/ ritonavir and dasabuvir, most studies only describe minor adverse effects in general population, significantly fewer than those encountered when using peg-interferon or first generation NS3/ 4A protease inhibitors [22, 23]. This drug combination is generally well tolerated regardless of the CKD stage [24]. Moreover, their safety profile in subjects with stage 3 CKD is comparable to that shown in patients with stage 1 or 2 CKD. However, when encountered, severe side effects were more frequent in patients with a higher degree of deterioration of the kidney function [24]. It is worth mentioning that, within the observed group, we did not asses any serious side effects, but only mild and moderate ones. To our knowledge, no study had described a connection between the incidence of minor adverse reactions and the comorbidities of the patient. We report a higher prevalence of asthenia, nausea and headache within diabetic and hypertensive patients. However, the presence of mild and moderate adverse effects did not represent a reason for discontinuing the antiviral therapy, in any of the cases.

None of the patients experienced worsening of kidney function. On the contrary, in most cases the use of DAAs and the achievement of SVR resulted in important amelioration of the renal function. Moreover, evidence found in previous similar studies state that both eGFR and serum creatinine improved by the end of treatment in patients with CKD stage 2 or 3, revealing the fact that this antiviral regimen does not produce alterations in renal function [24, 25].

In diabetic patients, the use of DAAs has been correlated with improved insulin sensitivity, leading to a satisfying glycemic control [26–28], a fact that has been noted within our group of HCV infected patients with renal impairment due to diabetes: those with poorly controlled glycemic levels achieved good control by the end of treatment, while most of the insulin-dependent subjects required a decrease in daily insulin doses. The hepatitis C viral core proteins interfere with the insulin receptor substrate-1 (IRS-1), increasing its degradation and blocking its bond with the insulin receptor [29–31]. The virus also affects insulin metabolism indirectly, by stimulating the production of interleukin-6 (IL-6) and tumor necrosis factor alpha (TNF-alpha) from sinusoidal liver cells; these cytokines have a role in enhancing the gluconeogenesis process [32].

Unlike the interferon-based regimens, the use of DAAs has been demonstrated to have minimal side effects in kidney transplant recipients [33–35]. In a large prospective observational cohort study, developed by Saxena et al., the use of paritaprevir/ ombitasvir/ ritonavir and dasabuvir proved its efficacy and safety in both kidney and liver transplant recipients [36]. However, due to possible interactions, the immunosuppressive therapy requires adjustments in the dosage and frequency of administration, while attentively monitoring the patient. Regarding this aspect, we reported the case of a kidney transplant recipient, who accidentally overlapped the two therapies (DAAs regimen and tacrolimus), therefore presenting to the hospital with severe hyperglycemia and very high levels of tacrolinemia [37]. The role of cytochrome P450 3A (CYP 3A) in paritaprevir metabolism explains the need of its co-administration with ritonavir *–* a CYP 3A inhibitor. On the other hand, tacrolimus is also metabolized by CYP 3A, being transported afterwards by the transmembrane P-glycoprotein. Both *C*YP 3A and the transmembrane P-glycoprotein are responsible of interfering with paritaprevir disposition [38]. It is important to mention that the use of interferon-free regimens based on paritaprevir/ ombitasvir/ ritonavir and dasabuvir in kidney transplant recipients was the only available option in our country at the moment, for this category of patients.

The prevalence of HCV in hemodialysis patients is notably higher than the general population, varying between 5 and 10% in Europe and in the USA [39]. In the Sub-Carpathian and South-Eastern regions of Romania, HCV seroprevalence was reported to be as high as 39.26% in hemodialyzed patients [40]. The development of DAAs has enlarged therapeutic options in patients with severe CKD and replacement of kidney function. Several studies have reported the safety and efficacy of paritaprevir/ ombitasvir/ ritonavir and dasabuvir for genotype 1b chronic hepatitis C patients undergoing dialysis due to ESRD [41–44]. In our study, all patients with hemodialysis completed the antiviral therapy, achieving SVR, with no severe reactions noted. However, HCV eradication may present disadvantages in kidney transplant candidates, as it is responsible for a longer time on the wait-list [45]. Many kidney transplant centers in the USA have recently adopted a different approach, that consists in offering the HCV-positive KT candidates allografts from HCV positive donors. This procedure would allow physicians to postpone HCV therapeutic measures until after the kidney transplant, by expanding donors’ criteria at the same time [46, 47]. The use of DAAs in HCV infected patients undergoing hemodialysis is imperative in subjects that are ineligible for kidney transplant, as well as in patients with advanced liver fibrosis that are also listed for liver transplant (in this case, obtaining HCV eradication could potentially avoid the need for liver transplantation) [48].

Regarding the association between SLE and HCV, there are several aspects that deserve to be mentioned, since our study included 5 patients with both pathologies. In the setting of SLE, viruses (including HCV) may play a triggering role. Moreover, hypocomplementemia, antinuclear antibodies (ANAs) and anticardiolipin autoantibodies (anti-CLAbs) are present in both SLE and HCV infection. The extrahepatic manifestations described in HCV-infected patients (such as arthralgia, myalgia, sicca syndrome) may, sometimes, be mistaken by a rheumatic disease (especially SLE) [49, 50]. A possible causality relation between the two pathologic entities can be sustained by a higher prevalence of HCV in patients with lupus (11%) compared to blood donors (1%), as well as frequent liver involvement in patients with SLE and HCV [50]. It is also probable that the altered immune response in individuals diagnosed with SLE could facilitate HCV infection [51]. Also, several studies suggest that HCV may be implicated in triggering the renal manifestations in SLE patients [49–51]. In chronic HCV infection, an increase of serum B-Lymphocyte activating factors may induce continuous B-cell activation and, thus, production of autoantibodies, which participate in the formation of immune complexes within the kidney; the consequence resides in the development of lupus nephritis [52].

## Conclusions

In patients with HCV and CKD, the combination of paritaprevir/ ombitasvir/ ritonavir and dasabuvir has shown promising effects. In the population we observed, the use of these direct acting antivirals in patients with CKD has demonstrated SVR rates similar to those seen in patients without CKD. Also, adverse effects were comparable to those seen in patients without renal impairment. No dose adjustments were made and no discontinuation of treatment was necessary. Notably, all 232 patients achieved SVR.

However, additional research and information are required, in order to assess efficacy, tolerability and adverse effects of these drugs and to completely clarify their impact on the natural history, as well as on the consequences of renal disease in HCV-infected patients.

## Data Availability

The datasets generated and/or analyzed during the current study are not publicly available. Data will not be shared due to intellectual property/ confidentiality issues, but are available from the corresponding author on reasonable request.

## Abbreviations

ANAs: Antinuclear antibodies
anti-CLAbs: Anticardiolipin autoantibodies
CKD: Chronic kidney disease
cytochrome CYP 3A: P450 3A
DAAs: Direct acting antivirals
DSV: Dasabuvir
eGFR: Glomerular filtration rate
EOT: End of treatment
ESRD: End-stage renal disease
HBV: Hepatitis B virus
HCV: Hepatitis C virus
HD: Hemodialysis
HIV: Human immunodeficiency virus
IL-6: Interleukin-6
IRS-1: Insulin receptor substrate-1 (IRS-1)
KT: Kidney transplantation
MPGN: Membrano-proliferative glomerulonephritis
OBV: Ombitasvir
PTV: Paritaprevir
r: Ritonavir
RNA: Ribonucleic acid
SLE: Systemic lupus erythematous
SVR: Sustained virologic response
TLRs: Toll-like receptors
TNF-alpha: Tumor necrosis factor alpha
